# The ‘energy revolution’ calls for technological innovation

**DOI:** 10.1093/nsr/nwac117

**Published:** 2022-06-22

**Authors:** Xinhe Bao

**Affiliations:** President of University of Science and Technology of China, and Senior Investigator at Dalian Institute of Chemical Physics, Chinese Academy of Sciences; China Editorial Board Member of NSR

Carbon neutralization symbolizes the ‘energy revolution’ in China, and has become a national strategic goal. More than 80% of carbon emissions in China come from energy utilization, especially the use of fossil energy. Therefore, the key to the ‘energy revolution’ lies in two areas: on the one hand, optimization of industrial structure and improvement of energy intensity, so as to achieve continuous reduction in total energy consumption while maintaining GDP growth; on the other hand, adjusting the consumption structure of primary energy and reducing the proportion of high-emission fossil energy, so as to reduce carbon dioxide emissions under the premise of meeting the energy needs of the whole society. The goal is to achieve carbon neutralization by 2060.

There are two major characteristics of energy in China [1]: first, structurally, it still relies heavily on fossil energy; second, the resource endowment is ‘poor oil, lean gas, and rich coal’. Petroleum and natural gas depend heavily on imports. In 2021, the import rates of oil and natural gas reached 72% and 45%, respectively, which imposes a great threat to energy security. Furthermore, there are 1.5 trillion tons of proven coal reserves in China, and 244 billion tons of technically mineable reserves, which can be used safely for 100 years. Therefore, based on the resource endowment of the nation, the strategy of ‘energy revolution’ should aim to achieve the clean and efficient utilization of coal, to increase the production of new energy, and to promote the optimal integration of fossil energy and new energy.

**To achieve clean and efficient utilization of coal.** In addition to improving the efficiency of coal-fired power generation through innovative technology, an important aspect of the optimal use of coal in China is to achieve efficient and clean conversion of coal for production of oil products and chemicals, so as to ensure energy and resource security. Traditional coal conversion technology involves a process of coal gasification giving synthesis gas (so called ‘syngas’, i.e. a mixture of CO/H_2_) followed by catalytic synthesis, i.e. Fischer-Tropsch synthesis (FTS), which was invented by two German scientists at the beginning of the last century. In this technology, hydrogen required for FTS is obtained from water through the water-gas-shift (WGS) reaction, which not only consumes energy, but also releases a large amount of carbon dioxide. Typically, ∼10 tons of carbon dioxide are emitted for the production of 1 ton of product (such as liquid fuels and olefins). Therefore, the research direction of coal conversion in the future should be focused on technologies to convert syngas to oxygen-containing compounds, thus retaining the oxygen atoms from the feedstock gas as much as possible in the final product. It is as urgent, if not more urgent, to optimize the process by shortening the process and improving the efficiency (e.g. product selectivity) [2]. But it would be most ideal to develop innovative technologies that utilize high-performance catalysts to directly ‘cut’ coal molecules into desired product molecules, in order to realize precise ‘molecular coal refining’.

**To improve the efficiency of new energy production.** From a technical point of view, the key should be high-performance solar cells and efficient electrochemical energy storage. Through wide efforts, significant progress has been made in solar cells, owing to many generations of new materials, from polysilicon to composite materials and crystalline silicon. However, the efficiency of converting solar energy into electricity is still limited to around 25%. There is great potential for development in the future [3]. Tandem solar cells, such as those composed of perovskite films (e.g. ∼1.7 eV) and crystalline silicon (e.g. ∼1.1 eV), and infrared quantum dot films (CQDs, e.g. <1 eV), are able to effectively convert wide-spectrum solar energy [4]. Therefore, breakthroughs are expected in many aspects relating to advanced materials and novel processes. Furthermore, in order to overcome the weaknesses of renewable energy sources, such as spatial distribution, low density and lack of continuity, development of high-efficiency energy storage devices is essential. Although pumped hydro storage will still be widely used in centralized, large-scale energy storage, chemical batteries will be a good alternative energy storage method that will match decentralized power generation devices. In this regard, high-safety solid-state lithium-ion batteries, high-performance lithium-sulfur batteries and lithium-air batteries will have great prospects. In addition, sodium batteries, aluminum batteries and large-capacity flow batteries that do not rely on lithium resources will also be important and shall be developed. At the same time, the recycling of used batteries has attracted wide attention and a breakthrough is also expected in the near future.

**To promote the optimal integration of fossil energy and renewable energy.** As an important fossil resource, coal has two main functions. One is to serve as an energy source, which reacts with oxygen to release energy, usually for heating and power generation. The other function is to be used as a reducing agent to free the metal from its oxide compounds, that is, metal smelting. Both processes produce large amounts of carbon dioxide. On the other hand, for new energy sources such as solar energy, wind energy, hydro energy and nuclear energy, the ultimate energy form is electricity. Therefore, direct utilization of renewable electricity for efficient heating will be an important research direction in the future. In this regard, promising technologies include high-temperature plasma, microwaves and electrical induction (superconducting induction). Electro-reduction processes have been widely used in metallurgical processes (e.g. electrolytic aluminum and copper). To improve efficiency, it is desirable to develop high-temperature electrochemical processes. Using electricity generated by renewable energy to produce hydrogen via the process of water electrolysis, and widely replacing carbon-based energy with hydrogen, will be essential to couple fossil energy and renewable energy in the future. Efficient, cheap and large-scale water electrolysis is a huge opportunity but remains a challenge. Technological breakthroughs will rely on high-efficiency membrane electrolysis (including proton membrane and alkaline membrane) and high-temperature inorganic membrane electrolysis, among others. This involves interdisciplinary scientific problems in both fundamental and applied fields of physics, chemistry, advanced materials and manufacturing. At the same time, large-scale, long-distance transport of hydrogen remains a challenge. Pipeline transportation has its advantages, but in addition to the need for innovative pipeline materials to overcome the problem of high-pressure hydrogen embrittlement, the economics of long-distance transportation (>1000 km) must also be carefully assessed. Other approaches currently under consideration include on-site chemical conversion of hydrogen into transportable liquid such as methanol and ammonia. There are no major technical barriers to these approaches, but technological advancements are still required to improve the economics and flexibility of the process. This again relies on substantial scientific progress and technological innovation.

In summary, to promote the ‘energy revolution’ and to achieve the goals of a carbon emissions peak and carbon neutrality in China, we must base all future development on the endowment of our energy resources, and strive to promote the combination of advanced energy technology with modern intelligent technology, new materials and advanced manufacturing technology. For the attainment of this goal, the optimal utilization of fossil energy is the basis, the scale of renewable energy is the key, the development of hydrogen energy technology is the focus and negative carbon technologies, including CCS (carbon capture and storage) and CCUS (carbon capture, utilization and storage) is the bottom line.
